# Effect of wood smoke exposure on vascular function and thrombus formation in healthy fire fighters

**DOI:** 10.1186/s12989-014-0062-4

**Published:** 2014-12-09

**Authors:** Amanda L Hunter, Jon Unosson, Jenny A Bosson, Jeremy P Langrish, Jamshid Pourazar, Jennifer B Raftis, Mark R Miller, Andrew J Lucking, Christoffer Boman, Robin Nyström, Kenneth Donaldson, Andrew D Flapan, Anoop SV Shah, Louis Pung, Ioannis Sadiktsis, Silvia Masala, Roger Westerholm, Thomas Sandström, Anders Blomberg, David E Newby, Nicholas L Mills

**Affiliations:** BHF Centre for Cardiovascular Science, University of Edinburgh, Edinburgh, UK; Department of Public Health and Clinical Medicine, Umeå University, Umeå, Sweden; ELEGI/Colt Laboratories, University of Edinburgh, Edinburgh, UK; Thermochemical Energy Conversion Laboratory, Umeå University, Umeå, Sweden; Edinburgh Heart Centre, Royal Infirmary of Edinburgh, Edinburgh, UK; Department of Analytical Chemistry, Stockholm University, Stockholm, Sweden

**Keywords:** Wood smoke, Air pollution, Fire-fighters, Vascular function, Thrombosis

## Abstract

**Background:**

Myocardial infarction is the leading cause of death in fire fighters and has been linked with exposure to air pollution and fire suppression duties. We therefore investigated the effects of wood smoke exposure on vascular vasomotor and fibrinolytic function, and thrombus formation in healthy fire fighters.

**Methods:**

In a double-blind randomized cross-over study, 16 healthy male fire fighters were exposed to wood smoke (~1 mg/m^3^ particulate matter concentration) or filtered air for one hour during intermittent exercise. Arterial pressure and stiffness were measured before and immediately after exposure, and forearm blood flow was measured during intra-brachial infusion of endothelium-dependent and -independent vasodilators 4–6 hours after exposure. Thrombus formation was assessed using the *ex vivo* Badimon chamber at 2 hours, and platelet activation was measured using flow cytometry for up to 24 hours after the exposure.

**Results:**

Compared to filtered air, exposure to wood smoke increased blood carboxyhaemoglobin concentrations (1.3% versus 0.8%; P < 0.001), but had no effect on arterial pressure, augmentation index or pulse wave velocity (P > 0.05 for all). Whilst there was a dose-dependent increase in forearm blood flow with each vasodilator (P < 0.01 for all), there were no differences in blood flow responses to acetylcholine, sodium nitroprusside or verapamil between exposures (P > 0.05 for all). Following exposure to wood smoke, vasodilatation to bradykinin increased (P = 0.003), but there was no effect on bradykinin-induced tissue-plasminogen activator release, thrombus area or markers of platelet activation (P > 0.05 for all).

**Conclusions:**

Wood smoke exposure does not impair vascular vasomotor or fibrinolytic function, or increase thrombus formation in fire fighters. Acute cardiovascular events following fire suppression may be precipitated by exposure to other air pollutants or through other mechanisms, such as strenuous physical exertion and dehydration.

**Trial registration:**

ClinicalTrials.gov Identifier: NCT01495325.

**Electronic supplementary material:**

The online version of this article (doi:10.1186/s12989-014-0062-4) contains supplementary material, which is available to authorized users.

## Background

Cardiovascular events are the leading cause of occupational death amongst fire fighters and account for approximately 45% of fatalities per year [1]. Moreover, the risk of acute myocardial infarction is increased 12- to 136-fold during fire suppression duties as compared to non-emergency duties and is likely to reflect a combination of factors including extreme physical exertion, mental stress, and exposure to heat and air pollutants [2]. Firefighters, during active fire suppression, are usually protected from smoke exposure by self-contained breathing apparatus (SCBA), however this is often disregarded in potentially hazardous, but tolerable situations, such as wildland fires where the long duration and remote location of fire fighting often renders SCBA wearing impractical [3]. Respiratory protection, therefore, often takes the form of a cotton rag or bandana tied around the nose and mouth.

Air pollution is an established risk factor for the development of both acute and chronic cardiovascular diseases [4-11] with exposure to particulate matter (PM) consistently associated with adverse cardiovascular health effects. The mechanisms through which specific air pollutants, and in particular traffic-derived air pollutants, influence the cardiovascular system have been intensively studied and an understanding of their effects on the pathophysiology of disease is emerging. In contrast, the health effects of wood smoke and biomass exposure have received little attention. Wood smoke contributes large quantities of ultrafine particles to our environment through the combustion of biomass for heating and cooking, and during major wildland fires. Firefighters have significant and often prolonged exposures during wildland fire fighting, an important duty of the fire service.

We have previously demonstrated that exposure to diesel exhaust impairs endothelial vasomotor and fibrinolytic function and increased ex-vivo thrombosis in man [12,13]. We have also demonstrated that exposure to wood smoke causes transient increases in arterial stiffness in healthy volunteers [14]. We therefore hypothesised that exposure to wood smoke, rich in ultrafine particulate matter, would have similar adverse effects and may explain the association between fire suppression and excess cardiovascular death. We therefore assessed the effect of exposure to wood smoke on vascular vasomotor and fibrinolytic function, and thrombus formation in healthy fire fighters.

## Results

Exposures were well tolerated with no adverse symptoms reported and all subjects completed both study visits.

**Figure 1:**
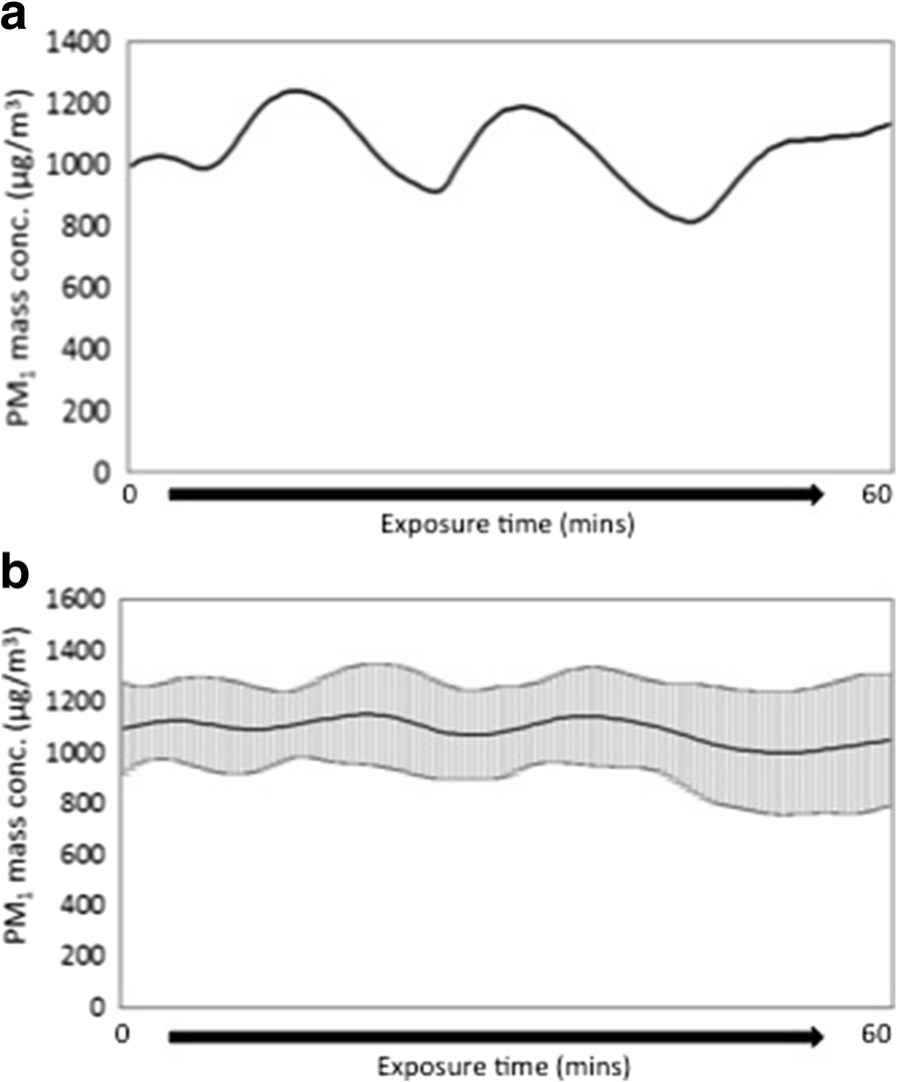
**Particulate matter concentration during exposures. (a)** A typical time-series of particle mass concentrations (PM_1_) in the chamber during a single 1 hour exposure measured with TEOM with data points every 30 seconds. **(b)** Average mass concentrations (PM_1_) in the chamber during the 1 hour exposures measured with TEOM (n = 16) every 30 seconds (mean ± standard deviation).

Within the chamber, particulate matter with an aerodynamic diameter <1 μm (PM_1_) concentrations were 1,115 ± 151 μg/m^3^, with nitrogen oxides (NO_x_) and carbon monoxide (CO) concentrations of 0.6 ± 0.1 ppm and 16.0 ± 1.1 ppm respectively (Table 1; Figure 1). Total PM mass was consistent with the target concentration for the study, as shown by both TEOM and filter measurements. The high EC/TC ratio illustrates the high soot content in wood smoke. The total PAH concentration in the exposure chamber was 4.3 ± 2.5 μg/m^3^, of which 90% was associated with wood smoke PM. The concentration of PM associated benzo[a]pyrene was 443 ± 302 ng/m^3^. The most abundant PAH compounds in the wood smoke PM fraction, accounting for 88 ± 1% of the total analyzed PAH (both PM associated and gas phase), were (in descending order): benzo[a]pyrene, chrysene, benzo[b]fluoranthene, benz[a]anthracene, benzo[e]pyrene, benzo[ghi]perylene, benzo[ghi]fluoranthene, indeno[1,2,3-cd]pyrene, pyrene, fluoranthene, benzo[k]fluoranthene and coronene (Additional file 1: Table S1).

**Table 1:** **Characterization of wood smoke exposure and particulate matter**

	**n**	**Unit**	**Mean**	**SD**	**Min**	**Max**
**PM**_**1**_ **mass concentration (TEOM)**	16	μg/m^3^	1,115	151	922	1,561
**PM**_**1**_ **mass concentration (filter)**	16	μg/m^3^	899	100	726	1,105
**Carbon monoxide**	16	ppm	16	6	8	25
**Nitrogen oxides**	16	ppm	0.6	0.3	0.3	1.0
**Elemental carbon/total carbon** ^**1**^	6	ratio	0.80	0.02	0.79	0.83
**Organic fraction of total PM** ^**1,2**^	6	%	23.1	4.7	16.9	28.8
**Soot fraction of total PM** ^**1**^	6	%	60.1	15.7	39.9	78.8
**PAH – PM associated (filter)** ^**1,3**^	6	μg/m^3^	3.9	2.3	1.5	6.7
**PAH – semi-volatile (PUF)** ^**1,3**^	6	μg/m^3^	0.4	0.3	0.1	0.9

^1^OC-EC and PAH analysis from selected samples throughout the campaign (n = 6).

^2^Estimated based on the OC-EC analysis (assuming a factor of 1.8 used to covert OC to total organic PM and a factor of 1.1 used to convert EC to total soot PM).

^3^Includes the PAHs: phenanthrene, anthracene, 4*H*-cyclopenta[def]phenanthrene, 2-phenylnaphthalene, fluoranthene, pyrene, 1-methylfluoranthene, benz[a]fluorene, benz[b]fluorene, 2-methylpyrene, 4-methylpyrene, 1-methylpyrene, benzo[c]phenanthrene, benzo[ghi]fluoranthene, benzo[b]naphtho[1,2-d]thiophene, benz[a]anthracene, chrysene, 3-methylchrysene, 2-methylchrysene, 6-methylchrysene, 1-methylchrysene, benzo[b]fluoranthene, benzo[k]fluoranthene, benzo[e]pyrene, benzo[a]pyrene, perylene, indeno[1,2,3-cd]fluoranthene, indeno[1,2,3-cd]pyrene, dibenz[a,h]anthracene, picene, benzo[ghi]perylene, dibenzo[a,l]pyrene, dibenzo[a,e]pyrene, coronene, dibenzo[a,i]pyrene and dibenzo[a,h]pyrene.

**Figure 2:**
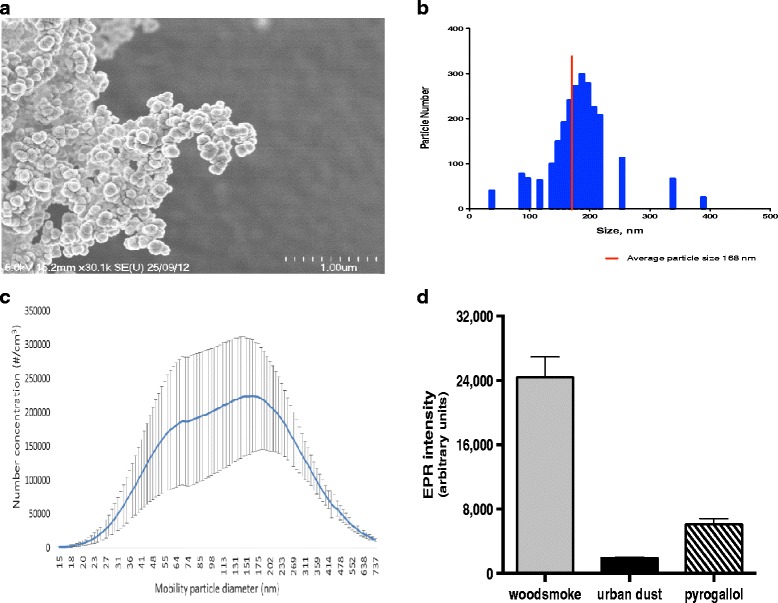
**Characterization of wood smoke particulate matter. (a)** Scanning electron microscopy (SEM) image of wood smoke particles. **(b)** Size distribution graph of the particle size as assessed by photon correlation spectroscopy with the mean primary particle size indicated by the red line. **(c)** Average particle number size distribution in the exposure chamber, measured by SMPS system. The plot displays the distribution as mean and standard deviation from all 16 exposures. Previous studies demonstrate the 50–80 nm peak consists of alkali salt particles (e.g. potassium sulphate and potassium chloride) and the 150–200 nm peak soot particles with more organic material [15,16]. **(d)** Electron paramagnetic resonance (EPR) signal intensity showing oxygen free radical generation from wood smoke particulates in the presence of the superoxide-selective spin-trap Tempone-H. Particulates from exposures collected on Teflon filters suspended in physiological saline solution at a concentration of 100 μg particles/mL. The standard reference material urban dust (100 μg particles/mL) and pyrogallol (100 μM) were used as controls. Data expressed as mean ± SEM (n = 4–5).

The mean primary particle size was 168 nm and the number size distribution (mobility diameter) was clearly bi-modal with a lower peak around 50–80 nm and an upper peak around 150–200 nm (Figure 2). Wood smoke particulate generated superoxide free radicals in physiological saline solutions in the absence of cells or tissue. Comparing equivalent masses of particulate, the superoxide generating capacity of wood smoke particulates was greater than standard reference material urban dust or pyrogallol controls (P < 0.001) (Figure 2).

### Haematology

Blood carboxyhaemoglobin concentrations increased from 0.9 ± 0.04 to 1.3 ± 0.04% immediately following exposure to wood smoke (P < 0.001) (Table 2). Total leucocyte, lymphocyte, neutrophil and platelet counts, were unaffected for up to 24 h after the exposure.

**Table 2:** **Haematological effects of exposure to wood smoke and filtered air**

		**Filtered air**	**Wood smoke**	**P value** ^**1**^
**Carboxyhaemoglobin, %**	Baseline	0.9 ± 0.0	0.9 ± 0.0	<0.001
	2 hours	0.8 ± 0.0	1.3 ± 0.0*	
	24 hours	0.8 ± 0.0	0.8 ± 0.0	
**Leucocytes × 10** ^**9**^ **cells/L**	Baseline	5.5 ± 0.4	5.6 ± 0.4	0.22
	2 hours	6.1 ± 0.3	6.4 ± 0.4	
	6 hours	6.5 ± 0.3	6.9 ± 0.4	
	24 hours	5.4 ± 0.3	5.6 ± 0.4	
**Lymphocytes × 10** ^**9**^ **cells/L**	Baseline	2.0 ± 0.2	2.0 ± 0.2	0.11
	2 hours	1.9 ± 0.1	1.8 ± 0.1	
	6 hours	2.0 ± 0.1	2.1 ± 0.1	
	24 hours	2.0 ± 0.2	2.0 ± 0.2	
**Neutrophils × 10** ^**9**^ cells/L**	Baseline	2.8 ± 0.3	2.9 ± 0.3	0.20
	2 hours	3.6 ± 0.3	3.9 ± 0.4	
	6 hours	3.8 ± 0.3	4.1 ± 0.3	
	24 hours	2.8 ± 0.2	3.0 ± 0.3	
**Platelets × 10** ^**9**^ cells/L**	Baseline	225 ± 6	229 ± 7	0.07
	2 hours	212 ± 6	219 ± 7	
	6 hours	205 ± 5	203 ± 9	
	24 hours	225 ± 7	234 ± 6	

Values are reported as mean ± SEM.

^1^2-way ANOVA with repeated measures comparing filtered air and wood smoke exposures.

*P <0.001 following Bonferroni correction comparing filtered air and wood smoke.at a given time point.

**P < 0.05 for trend across time.

### Arterial stiffness and vascular function

Resting blood pressure and heart rate were unchanged during either exposure or for up to 24 h after exposure (Table 3). Augmentation index, augmentation pressure and pulse wave velocity increased immediately after exposure (P > 0.05), but changes were similar following exposure to wood smoke and filtered air (P > 0.05 for all comparisons of wood smoke versus filtered air).

**Table 3:** **Haemodynamic effects of exposure to wood smoke and filtered air**

		**Baseline**	**Post-exposure**										
			**0 mins**	**10 mins**	**20 mins**	**30 mins**	**40 mins**	**50 mins**	**1 hr**	**2 hrs**	**6 hrs**	**24 hr mean**	**P-value** ^**1**^
Systolic pressure, mmHg	Filtered air	135 ± 2	134 ± 2	133 ± 2	130 ± 3	131 ± 2	131 ± 2	130 ± 3	133 ± 2	132 ± 2	130 ± 2	124 ± 2	0.59
	Wood smoke	132 ± 2	135 ± 3	131 ± 3	130 ± 3	131 ± 3	130 ± 3	130 ± 3	135 ± 4	134 ± 5	131 ± 3	127 ± 2	
Diastolic pressure, mmHg	Filtered air	75 ± 2	74 ± 3	75 ± 2	75 ± 2	73 ± 4	76 ± 2	76 ± 2	76 ± 2	76 ± 2	76 ± 27	68 ± 2	0.89
	Wood smoke	75 ± 2	74 ± 2	74 ± 2	73 ± 2	74 ± 2	74 ± 2	76 ± 2	77 ± 2	77 ± 2	75 ± 2	70 ± 2	
Heart rate, bpm	Filtered air	63 ± 2	63 ± 3	63 ± 3	61 ± 3	60 ± 2	59 ± 2	59 ± 2	58 ± 2	58 ± 2	59 ± 2	56 ± 2	0.12
	Wood smoke	61 ± 3	63 ± 3	63 ± 3	62 ± 3	62 ± 3	61 ± 3	61 ± 3	60 ± 3	60 ± 3	58 ± 3	55 ± 2	
∆ Augmentation pressure, mmHg	Filtered air	-	0.2 ± 0.5	−0.4 ± 0.7	−0.7 ± 0.6	−0.1 ± 0.7	−0.2 ± 0.7	0.1 ± 0.7	0.7 ± 0.7	-	-	-	0.90
	Wood smoke	-	1.0 ± 0.8	−0.2 ± 0.3	−0.5 ± 0.3	−0.7 ± 0.4	−0.3 ± 0.5	0.6 ± 0.5	0.2 ± 0.5	-	-	-	
∆ Augmentation index @75 bpm, %	Filtered air	-	0.01 ± 4.6	−1.7 ± 6.7	−3.9 ± 6.1	−2.8 ± 7.6	−3.0 ± 7.5	−2.2 ± 7.0	−1.2 ± 7.2	-	-	-	0.72
	Wood smoke	-	2.3 ± 7.6	−1.1 ± 4.0	- 2.0 ± 5.1	−3.2 ± 5.0	−3.0 ± 6.4	−1.3 ± 6.7	−1.6 ± 6.0	-	-	-	
∆ Pulse wave velocity, m/s	Filtered air	-	0.1 ± 0.1	0.0 ± 0.1	0.1 ± 0.1	0.0 ± 0.1	−0.1 ± 0.1	0.0 ± 0.1	0.4 ± 0.5	-	-	-	0.98
	Wood smoke	-	0.0 ± 0.1	−0.1 ± 0.1	0.0 ± 0.1	−0.1 ± 0.1	0.0 ± 0.1	−0.1 ± 0.1	0.1 ± 0.1	-	-	-	

Values are reported as mean ± SEM.

^1^2-way ANOVA with repeated measures comparing wood smoke and filtered air (baseline to 6 hrs); paired Students t-tests were performed for 24 hr means (P > 0.05 for all).

**Figure 3:**
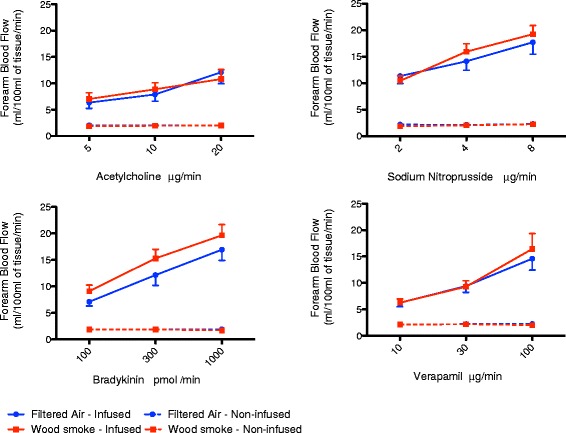
**Effect of wood smoke and filtered air on forearm blood flow.** There was a dose-dependent increase in forearm blood flow with each vasodilator (2-way ANOVA with repeated measures, P < 0.01 for all), however there were no differences in blood flow response to acetylcholine (P = 0.91), sodium nitroprusside (P = 0.52) or verapamil (P = 0.63) between exposures. In contrast, there was an increase in the forearm blood flow to bradykinin following exposure to wood smoke compared to filtered air (P = 0.003). All data expressed as mean ± SEM. There are no differences in blood flow in the non-infused arms and therefore these data points are overlaid.

**Figure 4:**
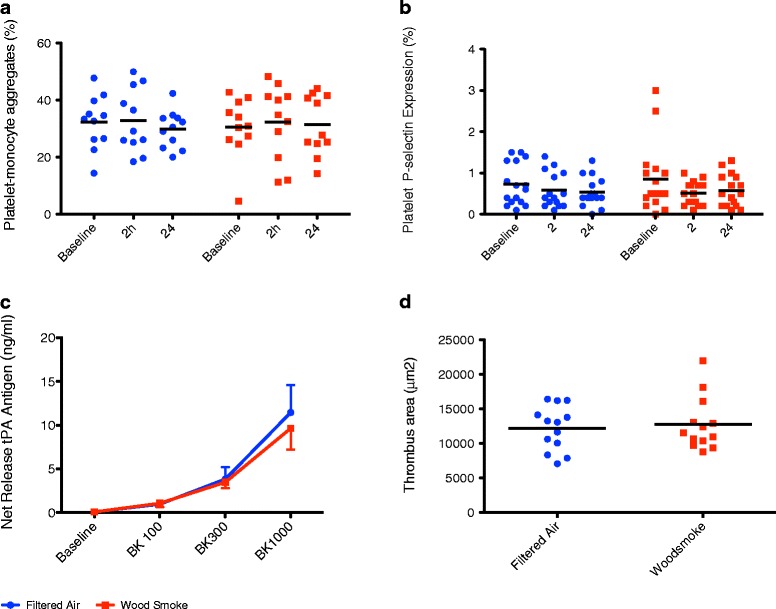
**Effect of wood smoke and filtered air on platelet activation, fibrinolysis and thrombus formation ex vivo. (a)** Platelet-monocyte binding and **(b)** platelet expression of P-selectin were unchanged 2 and 24 hours following exposure to wood smoke or filtered air (ANOVA with repeated measures, P > 0.05, n = 11–16). **(c)** Bradykinin caused a dose-dependent release of tissue-plasminogen activator (t-PA) antigen (2-way ANOVA with repeated measures, P < 0.01), which was similar after both exposures (P > 0.05, n = 16). **(d)** Thrombus formation under high-sheer conditions in the Badimon chamber was similar 2 hours after exposure to wood smoke or filtered air (Student’s *t*-test, P > 0.05, n = 13). All data expressed as mean ± SEM.

There was a dose-dependent increase in forearm blood flow with each vasodilator (P < 0.01 for all). However there were no differences in blood flow responses to acetylcholine (P = 0.91), sodium nitroprusside (P = 0.52) or verapamil (P = 0.63) between exposures (Figure 3). In contrast, there was an increase in forearm blood flow with bradykinin infusion following exposure to wood smoke compared to filtered air (P = 0.003). Bradykinin caused a dose-dependent release of tissue plasminogen activator antigen (P < 0.01), which was similar after both exposures (P = 0.72) (Figure 4).

### Platelet activation and thrombosis

Platelet-monocyte binding, monocyte surface expression of CD40 and platelet surface expression of CD40L and P-selectin were similar following wood smoke and filtered air exposure at 2 and 24 h following exposure (P > 0.05 for all) (Figure 4). There was no difference in thrombus formation following exposure to wood smoke compared with filtered air (thrombus area 12,216 ± 3,237 versus 12,775 ± 3,831 μm^2^, P = 0.54) (Figure 4).

## Discussion

Controlled exposure to wood smoke at high particulate concentrations does not impair endothelial-dependent or -independent vasodilation or increase thrombosis in firefighters. Using established methodology and a comprehensive assessment of cardiovascular health we found no adverse effects of wood smoke to explain the cardiovascular risk associated with fire suppression duties.

Whilst there have been no prior controlled exposures to wood smoke in firefighters, the effect of wood smoke on vascular function has been studied in healthy volunteers [17] and systemic inflammatory effects have been observed in firefighters responding to forest fires [18-20]. We have previously demonstrated that exposure to wood smoke for 3 hours at a lower PM concentration of 300 μg/m^3^ caused a transient increases in arterial stiffness and heart rate [14]. In contrast, we found no effect on arterial stiffness or heart rate following exposure to wood smoke at three-fold higher concentrations in firefighters over one hour. The overall dose was similar in both studies. These discordant findings may be explained by differences in susceptibility to wood smoke between healthy volunteers and firefighters with the latter having had multiple previous exposures to wood smoke through their occupation. There is some evidence that repeated exposure to smoke upregulates anti-oxidant levels in the airways and may diminish the effects of an acute exposure [21]. However we restricted enrollment to those firefighters who had not attended a major structural or wildland fire for more than one week prior to study visits and the effects of previous exposure on anti-oxidant levels are likely to be transient. It is also interesting to note that the baseline heart rates in our previous study [14] were approximately 10 bpm higher than in this study, perhaps suggesting previous participants were more susceptible to any effects of exposure on the autonomic nervous system. The duration of exposure may also be important with effects of wood smoke on arterial tone emerging after longer exposure periods. However, Forchhammer et al. recently observed no effect of wood smoke on peripheral arterial tone assessed by finger plethysmography despite delivering PM at 350 μg/m^3^ for up to 3 hours [17].

Venous occlusion plethysmography with intra-arterial infusion of vasodilators is widely regarded as the ‘gold-standard’ assessment of vascular function. We found no detrimental effect of exposure to wood smoke or filtered air on either endothelium-dependent or -independent vasodilatation. In fact we demonstrate a small increase in blood flow in response to bradykinin infusion following wood smoke exposure. It is plausible this was due to the effects of carbon monoxide exposure at concentrations that were sufficient to increase carboxyhaemoglobin concentrations and indeed, carbon monoxide is emerging as an important mediator of the vasodilator effects of bradykinin in the vessel wall [22]. The levels of carbon monoxide in this study were 4-fold higher than previous exposures to dilute diesel exhaust [23] and therefore it is plausible that vasodilation as a consequence of higher gaseous pollutants (carbon monoxide or nitrogen oxides [24]), may be important here. Nevertheless, it is unlikely that this would offset any detrimental effects of wood smoke PM on forearm blood flow across other vasodilators. There were no other important or adverse effects of wood smoke on vascular function, including endogenous fibrinolysis, platelet activation and thrombosis. Taken together these findings suggest that exposure to wood smoke is unlikely to be the primary cause of acute adverse cardiovascular events in firefighters.

Whilst traffic-related air pollution is an established trigger for acute myocardial infarction [4,6,9,25], there are few studies that have linked exposure to wood smoke or biomass with cardiovascular events. The risk in firefighters may be mediated by other factors, such as exposure to extreme heat, physical exertion and psychological stress. Heat stress results from both high ambient temperatures and exercise-induced metabolic activity, exacerbated by insulated protective clothing. In controlled studies, heat stress causes vasodilatation and fluid loss, resulting in a reduction in cardiac output and a hypercoagulable state [26-29]. Strenuous physical exertion is an independent trigger of sudden cardiovascular events, particularly in individuals unaccustomed to exercise [30]. Fire suppression often requires firefighters to work at the extremes of physical capability associated with heart rates in excess of age predicted maximums [26,27,31-33] and for long periods with shifts frequently lasting 12 to 24 h [34]. Whilst exposure to wood smoke may not in isolation cause vascular dysfunction or induce a prothrombotic state, it remains plausible that firefighters responding to wildland fires are at increased risk of an acute cardiovascular event through a combination of factors that could still include wood smoke. Furthermore, firefighters are also exposed to a heterogenous mix of air pollutants during other activities and although the use of breathing apparatus is employed in these situations, at the perimeter of such fires and in the aftermath when, breathing apparatus is removed important exposures may occur.

It is perhaps surprising that exposure to fine wood smoke particles at concentrations in excess of 1,000 μg/m^3^ had no adverse effects given that exposure to diesel exhaust at 300 μg/m^3^ has previously been shown to impair vascular function and increase thrombus formation in healthy men [12,13,35]. Differences in particle properties such as size, composition and surface chemistry between these exposures are likely to be important. Although the majority of wood smoke and diesel exhaust particles are in the ultrafine size fraction, the diameter of primary wood smoke particles was 5-fold larger than diesel exhaust particles (primary particle size of NIST standard reference material 2975 is 31 nm). Wood smoke particles are therefore perhaps less likely to deposit in terminal bronchioles or alveolar space and therefore to translocate or deliver soluble components into the circulation where they could directly effect the cardiovascular system. If wood smoke particles are unable to translocate due to larger size then they could perhaps cause a systemic inflammatory response, exerting late effects that were missed by undertaking assessments early after exposure. Others have shown that controlled exposure to fine and coarse PM is associated with early autonomic imbalance: rapid elevation of blood pressure and heart rate, and decreased heart rate variability immediately following exposure [36-39]. Conversely, we may have missed any immediate effects mediated by autonomic imbalance that were not comprehensively assessed in this study, although blood pressure and heart rate were unaffected acutely and over the 24 hour study period. Differences in surface chemistry are also likely to be important. Whilst wood smoke particles were able to generate super-oxide radicals, there were major differences in the PAH profile between wood smoke particles, where high molecular weight PAHs (≥228 Da) dominated, as compared to diesel exhaust particles [40].

There are some limitations to our study that merit consideration. The time points chosen to conduct our assessments post-exposure were based on the results of previous studies [12-14,23,24,35,41-46]. However, it is possible that wood smoke particles either exert an immediate or late effect on the cardiovascular system and we may have missed such effects. Additionally, the duration of exposure is also likely to be important with cumulative exposures over many days or weeks difficult to model experimentally. We recruited early career firefighters to minimize potential for confounding due to pre-existing vascular disease. Firefighters are exposed to complex mixtures of air pollutants derived from different sources, many of which may be more toxic than our simulated wildland fire exposure. It is plausible that firefighters with risk factors or subclinical disease would be more susceptible to any adverse cardiovascular effects of wood smoke. According to the widely recognized “healthy worker effect” in occupational medicine, it is common that susceptible individuals leave the workplace early due to symptoms, discomfort or acute illness. This may lead to selection bias with the remaining workers less sensitive or resistant to these noxious factors. Furthermore, we prospectively powered the study based on the measurements of primary end points made during previous studies [12,47-49]. Although we are confident that we have not missed effects on endothelial function or ex vivo thrombosis, we acknowledge that we may have had insufficient power to detect modest changes in some of the secondary end points, and thus cannot exclude the possibility of false-negative findings confounding their assessment. Nevertheless, even allowing for these limitations, in a carefully designed and controlled study with a comprehensive assessment of cardiovascular function, we found no adverse effects of exposure to wood smoke.

## Conclusions

Isolated wood smoke exposure at concentrations occurring in the vicinity of major wildland fires did not impair vascular vasomotor or fibrinolytic function, or increase thrombus formation in firefighters. The acute cardiovascular events associated with fire suppression may not be directly related to wood smoke exposure, rather they may be precipitated by other pollutants or mechanisms such as strenuous physical exertion and dehydration.

## Methods

### Subjects

Sixteen healthy non-smoking male volunteers (median age 26, range 21–26 years) were enrolled into the study. The study was performed with the approval of local research Ethics Committees, in accordance with the Declaration of Helsinki and the written informed consent of all volunteers. Firefighters were recruited using advertisements in local fire stations. Exclusion criteria were cigarette smoking or the use of snus (tobacco snuff), the use of regular medication (specifically non-steroidal anti-inflammatory drugs, vitamins or anti-oxidant supplements), known ischemic heart disease, arrhythmia, diabetes mellitus, hypertension, renal or hepatic impairment, asthma, or inter-current infection. Subjects had normal lung function and reported no respiratory symptoms in the 6-week period preceding the study. Subjects had no occupational fire exposure (wildland or structural) for a week preceding study visit.

### Study design

Subjects attended on two occasions at least one week apart and were exposed to filtered air or wood smoke for one hour in a double-blind randomized crossover design. Subjects attended at 8 am on the morning of study for initial bloods and for the fitting of Holter and ambulatory blood pressure monitors. Exposures were performed at 10 am in a dedicated exposure facility by researchers and technical staff not involved in the subsequent clinical assessment. Subjects remained indoors following exposure to minimize any confounding effects from ambient air pollution. Vascular studies were carried out in a quiet, temperature-controlled room maintained at 22°C to 24°C with subjects lying supine. All subjects abstained from alcohol and caffeine for 24 h, and from food for at least 4 h before each vascular study.

The primary endpoints were forearm blood flow, estimated t-PA release from the forearm circulation and ex-vivo thrombus formation. Secondary endpoints included arterial stiffness, platelet activation, and changes in haematological variables. Based on previous studies [12-14,23,24,35,41-46], pulse wave analysis and velocity were assessed immediately after exposure, a study of ex-vivo thrombus formation, Badimon study was performed at 2 h, and venous occlusion plethysmography undertaken 4 to 6 h after exposures to wood smoke and filtered air. Venous blood was sampled at baseline, 2, 6 and 24 h after each exposure for storage and quantification of carboxyhaemoglobin. Subjects were fitted with an ambulatory blood pressure monitor (Spacelabs 90217; Spacelabs, Healthcare Ltd, Hertford, UK) prior to each exposure and monitored for 24 h.

### Wood smoke exposure

Exposures were performed in a purpose-built exposure chamber in Umeå, Sweden. During each exposure, subjects performed moderate exercise (to generate an average minute ventilation of 20 L/min/m^2^) on a bicycle ergometer that was alternated with rest at 15-min intervals.

Wood smoke was generated using a common Nordic wood stove using birch wood in an incomplete combustion firing procedure (partial air-starved conditions), generating a soot-rich aerosol emission. To generate relatively constant incomplete combustion conditions during the 1 hour exposures, small batches (0.5–1.0 kg) birch wood logs were inserted every 5–15 minutes to maintain a high burn rate with repeated air-starved conditions. This procedure was in accordance with our previous wood smoke exposure study [14]. The birch wood was stored outdoors under roof cover for approximately 2.5 years before use and had a moisture content of 16–17% at the time of this study. The wood smoke was diluted with HEPA filtered air in three steps and continuously fed into and through a controlled environment exposure chamber (17 m^3^) to achieve a steady state concentration.

The atmosphere in the chamber was monitored for pollutants using continuous measurement of oxides of nitrogen (NOx) (chemiluminescence, CLD 700 Ecophysics, >0.001 ppm) and carbon monoxide (CO) (IR, UNOR6N Maihak). Fine (<1 μm) particulate matter (PM_1_) mass concentration was measured on-line using a tapered element oscillating microbalance (TEOM 1400, Thermo Scientific). Integrated with the TEOM a filter (Teflon) sampling line was used to determine the mass concentration gravimetrically. A CO alarm instrument (MC400, Monicon Technology) was used in the chamber during the exposures. The target PM_1_ concentration in the chamber was 1,000 μg/m^3^.

The equivalent mobility diameter (in the range 10–600 nm) of the wood smoke particles was measured in the chamber using a scanning mobility particle sizer (SMPS) (TSI GmbH). Organic (OC) and elemental carbon (EC) were determined using thermal-optical carbon analysis (according to the EUSAAR_2 protocol). These concentrations are regularly encountered at the perimeter of forest fires [50] and indoors when cooking with solid fuels [51], and are below the UK workplace 8 hour average exposure limits (HSE EH40 Workplace Exposure Limits 2005). The temperature in the chamber maintained between 21–24°C with a relative humidity of 50%.

### Polycyclic aromatic hydrocarbon analysis

The collected wood smoke PM, and polyurethane foam (PUF) plugs with sampled semi-volatile PAHs were extracted with pressurized fluid extraction using an ASE 200 Accelerated Solvent Extractor system (Dionex Corporation, Sunnyvale, CA, USA). Wood smoke PM was extracted with a solvent composition consisting of toluene and methanol 9:1 at 200°C and 3000 psi (20.7 MPa). PUFs were extracted with hexane at 110°C and 500 psi (3.45 MPa). Details on instrumental parameters are available elsewhere [52,53]. Solid phase extraction sample cleanup was performed to remove polar constituents from the samples according to Christensen et al. [54] followed by instrumental analysis using an automated high pressure liquid chromatography-gas chromatography–mass spectrometry system (HPLC-GC-MS) [40]. The HPLC part of the system was used for PAH fraction using the back flush technique, where PAHs with 3 and more rings were isolated and introduced into the GC-MS system for separation and detection. The MS was operated in selected ion monitoring mode, and the PAHs were identified using compound specific mass to charge ratio and relative retention time on the GC capillary column. In total 36 PAHs in the range of 178 – 302 Da were analyzed (Additional file 1: Table S1).

### Electron paramagnetic resonance

To provide a measure of particle reactivity EPR was used to establish oxygen-centred free radical generation from particulates collected from exposures (Langrish et al. [7]). A 1.6 mm diameter section of Teflon filter from the carbon analysis filter line of the exposure chamber and suspended in physiological saline solution (Krebs buffer, composition in mM: 118.4 NaCl, 25 NaHCO_3_, 11 glucose, 4.7 KCl, 1.2 MgSO_4_, 1.2 KH_2_PO_4_, 2.5 CaCl_2_) at a particle concentration of 100 μg/mL. Samples were vortexed for 1 min, followed by 30 min sonication sonication (100% power; Fisherbrand FB11002; Fisher Scientific, Loughborough, UK). Suspensions were incubated with the spin-trap, Tempone-H (1 mM; Enzo Life Sciences, Exeter, UK), immediately before the initial measurement. Tempone-H is a highly sensitive spin-trap that shows selectivity for superoxide, forming a stable product that can be measured by EPR [55]. The standard reference material urban dust (SRM1649a; National Institute of Standards and Technology, Gaithersburg, USA) was used as positive control particulate (note, that the results cannot be directly compared to filter particulates, as the proportion of the mass of wood smoke particulate unbound to the filter cannot be determined in the present study). Pyrogallol (100 μM) was used as a second positive control which spontaneously generates superoxide radicals in this buffer [56]. Samples were kept at 37°C throughout and measurements were taken after 30 and 60 min by drawing 50 μL of sample into a capillary tube (VWR International, Lutterworth, UK) and sealing with a plug of soft sealant (Cristaseal, VWR International). An X-band EPR spectrometer (Magnettech MS-200, Berlin, Germany) was used with the following parameters: microwave frequency, 9.3–9.55 Hz; microwave power, 20 mW; modulation frequency, 100 kHz; modulation amplitude, 1500 mG; center field, 3365 G; sweep width, 50 G; sweep time, 30 s; number of passes, 1. Baseline signals from blank (non-exposed) filters were subtracted from that of filters with particulate.

### Arterial stiffness

All measurements of arterial stiffness were performed at baseline, and at 10-min intervals after the exposure for one hour as previously described [14]. Pulse rate and blood pressure were measured using a validated semi-automated oscillometric sphygmomanometer (Boso-Medicus, Boso, Jungingen, Germany). Central arterial stiffness measured by pulse wave analysis was determined with a high-fidelity handheld tonometer (Millar Instruments, Texas, USA) at the right radial artery using the SphygmoCor™ system (AtCor Medical, Sydney, Australia). Carotid-femoral pulse wave velocity measurements were made using the Vicorder system (Skidmore Medical, UK).

### Vascular studies

All subjects underwent brachial artery cannulation with a 27-standard wire gauge steel needle under controlled conditions. After a 30-min saline infusion, acetylcholine at 5, 10, and 20 μg/min (endothelium-dependent vasodilator that does not release tissue plasminogen activator [t-PA]; Merck Biosciences); bradykinin at 100, 300, and 1000 pmol/min (endothelium-dependent vasodilator that releases t-PA; Merck Biosciences); sodium nitroprusside at 2, 4, and 8 μg/min (endothelium-independent vasodilator that does not release t-PA; David Bull Laboratories) and verapamil at 10, 30, and 100 μg/min (endothelium- and NO-independent vasodilator that does not release t-PA) were infused for 6 min at each dose. Vasodilators were separated by 20-min saline infusions and given in a randomized order except from verapamil, which was always given last due to its longer duration of action [57]. Forearm blood flow was measured in infused and non-infused arms by venous occlusion plethysmography with a mercury-in-silicone elastomer strain gauges as described previously [58].

Venous cannulas (17 gauge) were inserted into large subcutaneous veins of the ante-cubital fossae of both arms. Blood (10 mL) was withdrawn simultaneously from each arm at baseline and during infusion of each dose of bradykinin and collected into acidified buffered citrate (Stabilyte tubes, Biopool International). Samples were kept on ice before being centrifuged at 2000 *g* for 30 min at 4°C. Platelet-free plasma was decanted and stored at −80°C before assay. Plasma t-PA antigen and activity concentrations were determined by enzyme-linked immunosorbant assay (TECHNOZYM® t-PA Combi Actibind®, Technoclone, Austria). Hematocrit was determined by capillary tube centrifugation at baseline and during infusion of bradykinin at 1000 pmol/min.

### Flow cytometry

Samples were obtained at baseline, at 2 h immediately prior to the thrombosis study and at 24 h post exposure, and processed according to previously described protocols [59]. In brief, blood was taken from an ante-cubital vein using a 21-gauge cannula and anti-coagulated with D-phenylalanyl-Lprolyl-L-arginine chloromethylketone (75 μL; Cambridge Biosciences, UK). Samples were not analysed unless venesection achieved rapid and uninterrupted blood flow. Five minutes after sample collection, samples were stained with the following conjugated monoclonal antibodies: phycoerythrin (PE)-conjugated CD14 (Dako, Denmark), PE-conjugated CD62P, and PE-conjugated CD154 (Becton-Dickinson, UK); PE-conjugated CD40, fluorescein isothiocyanate (FITC)-conjugated CD42a, and FITC-conjugated CD14 (Serotec, USA); and appropriate control isotypes. All antibodies were diluted 1:20. Once stained, samples were incubated for 20 min at room temperature to identify P-selectin and CD40L on the platelet surface and CD40 on the monocyte surface. Platelet–monocyte samples were fixed with FACS-Lyse (Becton-Dickinson). Platelet samples were fixed with 1% paraformaldehyde. Samples were analysed within 24 h using a FACScan flow cytometer (Becton-Dickinson). Platelet–monocyte aggregates were defined as monocytes positive for CD14. Data analysis was performed using FlowJo (Treestar, USA).

### Ex-vivo thrombosis studies

Thrombus formation was measured using the Badimon chamber as previously described [13,23]. In brief, a pump was used to draw blood from an antecubital vein through a series of consecutive cylindrical perfusion chambers maintained at 37°C in a water bath. Carefully prepared strips of porcine aorta, from which the intima and a thin layer of media had been removed, acted as the thrombogenic substrate. Each study lasted for 5 min during which flow was maintained at a constant rate of 10 mL/min. Porcine strips with thrombus attached were removed and fixed in 4% paraformaldehyde, wax embedded, sectioned, and stained with Masson’s Trichrome. Images were acquired at × 20 magnification, and the thrombus area was measured using an Ariol image acquisition system (Leica Microsystems GmbH, Germany) by a blinded operator. Results from at least six sections were averaged to determine thrombus area for each chamber, as described previously [13,23].

### Data analysis and statistics

A sample size of 16 gives us >90% power to detect a 10% difference in thrombus area, 17% difference in mean t-PA release and 22% difference in forearm blood flow at a significance level of 5% [12,47-49]. Continuous variables are reported as mean ± standard error of the mean (SEM). Statistical analyses were performed with GraphPad Prism, version 5.0 (Graph Pad Software, USA) by 2-way analysis of variance (ANOVA) with repeated measures and 2-tailed Student *t-*test, where appropriate. Statistical significance was taken at two-sided *P* < 0.05.

## Additional file

Additional file 1: Table S1. Mean concentration of polyaromatic hydrocarbons (PAH).
