# Supplementary material for: Effect of wood smoke exposure on vascular function and thrombus formation in healthy fire fighters
Source: Part Fibre Toxicol. 2014 Dec 9;11:62. doi: 10.1186/s12989-014-0062-4 (PMC4338635; doi:10.1186/s12989-014-0062-4)
Supplement: Additional file 1: Table S1. — Mean concentration of polyaromatic hydrocarbons (PAH). [file 12989_2014_62_MOESM1_ESM.docx]

**Additional file 1: Table S1.** Mean concentration of polyaromatic hydrocarbons (PAH)

|  | PM  (ng/m^3^) | PUF  (ng/m^3^) |
| --- | --- | --- |
| Benzo(a)pyrene | 443 | 0 |
| Chrysene | 433 | 1 |
| Benzo(b)fluoranthene | 409 | 0 |
| Benz(a)anthracene | 353 | 1 |
| Benzo(e)pyrene | 300 | 0 |
| Benzo(ghi)perylene | 298 | 0 |
| Benzo(ghi)fluoranthene | 237 | 4 |
| Indeno(1,2,3-cd)pyrene | 223 | 0 |
| Pyrene | 204 | 92 |
| Fluoranthene | 201 | 102 |
| Benzo(k)fluoranthene | 197 | 0 |
| Coronene | 134 | 0 |
| Benzo(c)phenanthrene | 80 | 3 |
| Perylene | 58 | 0 |
| Dibenz(a,h)anthracene | 36 | 0 |
| Picene | 35 | 0 |
| Phenanthrene | 33 | 159 |
| Indeno(1,2,3-cd)fluoranthene | 32 | 0 |
| 1-Methylfluoranthene | 24 | 2 |
| 2-Methylchrysene | 23 | 1 |
| 1-Methylpyrene | 20 | 3 |
| 4-Methylpyrene | 17 | 5 |
| Dibenzo(a,e)pyrene | 16 | 0 |
| 6-Methylchrysene | 15 | 1 |
| Benz(a)fluorene | 14 | 2 |
| 1-Methylchrysene | 12 | 1 |
| 2-Phenylnaphthalene | 10 | 10 |
| Anthracene | 10 | 19 |
| 2-Methylpyrene | 10 | 3 |
| 3-Methylchrysene | 9 | 1 |
| Dibenzo(a,i)pyrene | 7 | 0 |
| Benz(b)fluorene | 6 | 2 |
| 4H-cyclopenta(d,e,f)phenanthrene | 3 | 8 |
| Dibenzo(a,l)pyrene | 2 | 0 |
| Dibenzo(a,h)pyrene | 2 | 0 |
| Benzo(b)naphto(1,2-d)thiophene | 0 | 0 |

PM: particulate matter, PUF: polyurethane foam
